# Interleukin-17 programs liver progenitor cell transformation into cancer stem cells through miR-122 downregulation with increased risk of primary liver cancer initiation

**DOI:** 10.7150/ijbs.70408

**Published:** 2022-02-21

**Authors:** Imène Gasmi, Camilia Machou, Aurélie Rodrigues, Arthur Brouillet, Trung Cong Nguyen, Benoit Rousseau, Adrien Guillot, Christophe Rodriguez, Vanessa Demontant, Yeni Ait-Ahmed, Julien Calderaro, Alain Luciani, Jean-Michel Pawlotsky, Fouad Lafdil

**Affiliations:** 1 Université Paris-Est, UMR-S955, UPEC, F-94000, Créteil, France.; 2 INSERM, U955, F-94000, Créteil, France.; 3 Institut Universitaire de France (IUF), Paris, F-75231 Cedex 05 France.; 4 Département de Pathologies, Hôpital Henri Mondor, Université Paris-Est, Créteil, France.; 5 Département de Virologie, Hôpital Henri Mondor, Université Paris-Est, Créteil, France.

**Keywords:** Liver progenitor cells, Interleukin-17, cancer stem cells, liver cancer, miR-122

## Abstract

Chronic inflammation is a key component in the development of virtually all types of primary liver cancers. However, how chronic inflammation potentiates or even may initiate liver parenchymal cell transformation remains unclear. Cancer stem cells (CSCs) represent an exciting target for novel anticancer therapeutic strategies in several types of cancers and were also described in primary liver cancers as tumor initiating cells. Recently, we reported a key role of Interleukin (IL)-17 in Liver Progenitor Cell (LPC) accumulation in preneoplastic cirrhotic livers. In this study, we evidenced *in vitro,* that long-term stimulation of LPCs with IL-17 led to their transformation into CSCs. Indeed, they acquired CSC-marker expression, and self-renewal properties, showed by their increased capacity to form spheroids. The miRNome analysis revealed that long-term IL-17 treatment of LPCs led to a 90% decrease in miR-122 expression. In a model using immunodeficient mice, ectopic engraftment of LPCs in an IL-17-enriched environment led to tumor occurrence with an aggressive phenotype. Contrastingly, in a murine model of hepatocellular carcinoma induced by a unique injection of diethyl-nitrosamine associated with chronic administration of carbon tetrachloride, IL-17-deficiency or anti-IL-17 therapy protected mice from liver tumor growth. In conclusion, we showed that a chronic exposure of LPCs to IL-17 cytokine promotes their transformation into CSCs. In addition, we demonstrated that IL-17-neutralizing strategies limit CSC occurrence and liver tumor progression through miR-122 restored-expression.

## Introduction

Primary liver cancer is the fifth-most frequent cancer worldwide and the second leading cause of cancer-related death 1. Hepatocellular carcinoma (HCC) accounts for 80% of all primary liver cancers and represents the leading cause of death in patients with chronic liver diseases. Intrahepatic cholangiocarcinoma (ICC) arising from bile ducts within the liver parenchyma is the second most common (15%) primary hepatic malignancy after HCC 2. Despite significant advances in liver cancer diagnosis and therapy over the past decade, the current anticancer treatments remain poorly effective in advanced stages of the disease 3.

HCC and ICC have been considered as distinct tumors that originate from different hepatic cells (i.e. hepatocytes vs biliary cells, respectively). However, emerging evidence suggest that both primary liver cancers could also originate from a common tumor initiating cell population 4, 5. Interestingly, Cancer Stem Cells (CSCs) have recently sparked the interest of scientists because of their capacity to initiate and enhance tumor progression of several cancers 6. CSCs have been found in virtually all types of cancers including HCC or ICC and their presence is generally associated with poor outcomes 7, which contribute to the failure of conventional targeted cancer therapies 8. However, the origin of CSCs, and the underlying mechanisms by which they develop in the liver remain unclear.

Liver Progenitor Cells (LPCs) are defined as intrahepatic quiescent cells that are activated in chronic liver diseases and participate to liver regeneration when the proliferation of remaining hepatocytes is impaired 9. They are bipotent cells that could differentiate into either hepatocytes or biliary cells. LPC accumulation, referred to as ductular reaction, is frequently observed particularly in cirrhotic livers with a worse prognosis 10. Interestingly, it has been observed that 28 to 50% of HCC express progenitor/stem cell markers 11. Besides, ICC is thought to originate from biliary cells in tight interaction with canals of Hering where the LPC compartment is located 12. For those reasons, it is accepted that LPCs have the potential to become tumor initiating cells because of their likelihood to transform into CSCs that ultimately evolve into heterogeneous lineages of cancer cells 5, 9, 13. Oncogenic processes leading to the transformation of LPCs into CSCs remain poorly understood.

Primary liver cancers occur in 90% of cases in an inflammatory context present in virtually all chronic liver diseases 14. The inflammatory response plays a key role in the activation of the LPC compartment 10, while chronic and persistent inflammatory response may favor LPC transformation into CSCs 5. In this inflammatory context, Th17 cells are crucial components among infiltrating immune cells in HCC 15 and numerous studies revealed that Interleukin-17A (IL-17), a cytokine mainly produced by Th17 cells, can directly act on stem cells to promote their proliferation, and thus, tissue repair. However, IL-17 may also participate in cancer initiation, tumor progression and metastasis 16. We recently reported a positive correlation between the number of IL-17-infiltrating cells and the number of LPCs accumulating in human cirrhotic livers from various etiologies 17. In addition, a high IL-17 expression has been associated with a poor prognosis of HCC 18, along with an increased hepatic tumor growth and metastasis 19. Recently, plasma IL-17 concentration has been identified as a predictor of subsequent HCC occurrence in liver cirrhosis patients 20, but whether and how IL-17 could play a role in the initiation of liver tumorigenesis remains unclear.

In this study, we hypothesized that IL-17 could participate in liver cancer initiation by promoting LPC transformation into a CSC phenotype. We showed *in vitro* that long-term exposure of LPCs to IL-17 leads to their transformation into CSCs, and we evidenced in two murine models that IL-17 neutralization limits cancer initiation and progression. These results suggest that IL-17-neutralizing strategies may be used to prevent primary liver cancer development.

## Materials and methods

### Human samples

Forty-five liver tissue samples from previously described patients 17 with diverse chronic liver diseases were analyzed. The study conformed to the ethical guidelines of the 1975 Declaration of Helsinki was approved by the local ethics committee Ile de France I (Institutional Review Board 2017‐A01215‐48) as required by the French legislation. Blocks of formalin‐fixed paraffin‐embedded samples from explanted livers were obtained from the Department of Pathology of Henri Mondor University Hospital (Creteil, France). For the assessment of CK19- , IL‐17-^-^ and CD133-positive cell density, slides were reviewed and scored by two independent evaluators, including a pathologist specialized in liver diseases, using a semi-quantitative score and patients were dichotomized into high versus low density of stained cells for each labeling as previously described 17.

### LPC cell lines and culture conditions

Bipotential Murine Oval Liver cells (BMOL) (kindly provided by Pr George C. Yeoh, School of Medicine and Pharmacology, University of Western Australia) and human HepaRG (obtained from Biopredic International, France) were cultured in a humidified atmosphere at 37 °C and 5% CO_2_ (with or without IL-17) for 10, 20, 30 and 40 days. Recombinant mouse and human IL-17 were purchased from R&D Systems. For BMOL culture, 50 ng/ml of recombinant mouse IL-17 was added every three days to William's medium supplemented with 5% Fetal Calf Serum (FCS), 10 µg/ml insulin and 10 ng/ml of recombinant mouse Epidermal Growth Factor (EGF). Fifty ng/ml of recombinant human IL-17 was added every 3 days to HepaRG cultured in William's medium supplemented with 10% FCS, insulin and hydrocortisone hemisuccinate at final concentration of 4 µg/ml and 50 µM respectively.

### Animals

#### Ectopic allograft of IL-17-expressing LPCs

For *in vivo* characterization of IL-17 tumorigenic effect, BMOL cells were genetically modified to constitutively express IL-17 and luciferase genes. A pUNO1-mIL17A plasmid (InvivoGen) was used in co-transfection experiments with the pGL4.51 (Luc2) vector (Promega) using the Lipofectamine® LTX Reagent with PLUS™ Reagent (Life Technologies), and stably expressing LPCs were isolated after 3 weeks of selection by G418 and blasticidin antibiotics. As an empty control vector, pUNO1-mcs plasmid was used. Subcutaneous implantation of 10^6^ transfected cells was performed into NOD/SCID mice in accordance with the local Committee of for the Use of Live Animals. *In vivo* cell expansion was monitored weekly for 12 weeks by bioluminescence. Briefly, mice were intraperitoneally injected with 200 µl of 15 mg/ml D-luciferin solution. Tumor size and cell density were assessed 10 minutes later using an IVIS spectrum imaging device (Perkin Elmer) under gas anesthesia (2.5% isoflurane). An auto-drawing feature was used to draw the region of interest (ROI) and the total number of photons emitted per second was calculated.

#### DEN+CCl_4_ murine model

C57BL/6J (Charles River) and C57BL/6J Il17a^tm1Yiw^/Il17a^tm1Yiw^ (kindly provided by Pr Yoichiro Iwakura) were injected intraperitoneally (i.p.) at 2 weeks of age with diethylnitrosamine (DEN, 25 mg/kg, Sigma Aldrich). From week 8 of age, only male C57BL/6J mice were injected twice per week with 10% carbon tetrachloride (CCl_4_), (2 mg/kg, Sigma Aldrich) for 14 weeks, and the last 6 weeks, mice were treated with 100 µg of anti-IL-17 antibodies or its isotype control (BE0173, BE0083, BioXcell) twice per week. Tumor areas were quantified using QuPath, a free open-source software for digital pathology analysis 21.

### Sphere forming assay

BMOL cells were cultured for 30 days in William's medium with 5% FCS (with or without IL-17 at 50 ng/mL) in adherence conditions. Then cells were collected and resuspended in serum-free DMEM/F12 media supplemented with 20 ng/ml of B27 supplement, recombinant mouse EGF and basic FGF. The cells were subsequently cultured in ultra-low attachment 12-well plates at a density of 5 cells/µL. To test BMOL self-renewal capacity, the spheres were counted and dissociated 7 days later with Accutase enzyme, then seeded again in ultra-low attachment wells at the same density of 5 cells/µL.

### Flow cytometry analysis 

For CD133 (Prominin-1) labeling, 1 million cells were resuspended in PBS supplemented with 2mM EDTA and 0.5% of bovine serum albumin. Anti-prominin-1 coupled with APC or IgG1 control antibodies (Miltenyi) were added in cell suspension and incubated for 15 min at 4 °C in the dark. Then the cells were washed and resuspended in PBS supplemented with EDTA and BSA for flow cytometry analysis.

### Immunostaining

Mouse tumors were fixed in 4% paraformaldehyde and embedded in paraffin. Sections were labeled with primary antibodies against CK-19 (TROMA III, DSHB), AFP (Santa Cruz, sc-8108), and CD133 (Abcam, ab19898) overnight at 4 °C. Then, sections were incubated for 45 min with goat anti-rat IgG Alkaline Phosphatase (MP-5404), donkey anti-goat IgG-B (SC-2042), or horse Anti-Rabbit IgG Peroxidase (Vector, MP-7401), respectively. Detection was performed using a liquid permanent red (Dako, K0640) for CK-19 staining. For AFP and CD133 immunostaining, a Vector ImmPRESS Reagent, Peroxidase with DAB exposure was used. HE and Red Sirius staining were performed in the Henri Mondor pathology department. Coloration staining and immunostaining quantification were performed using ImageJ software (NIH) by means of color deconvolution plugin followed by MRI Fibrosis Tool 22 for Sirius red coloration, and IHC profiler plugin for AFP and CD133 staining.

### RNA analysis

Total RNA was isolated from lysed cells using RNeasy Mini kit (Qiagen) according to the manufacturer's protocol. The RNA concentration in each sample was assessed using a Nanodrop ND-1000 spectrophotometer (Thermoscientific). Two µg of RNA was reverse transcribed from random primers using a High capacity kit (AB applied). *Cd133, Epcam, Aldh, Klf4, Gpc3, Afp, Cyclin D1, Cyclin E, P21, Zeb1, Snail, Ck19, Col1, Thy1, Cd44, Lgr5 and Αsma* transcript levels were measured by quantitative reverse transcriptase polymerase chain reaction (RT-qPCR) using a LightCycler 480 (Roche). DNA amplification was performed using 25 ng of cDNA, 10 μM forward and reverse primers and the Quantitect SYBR green PCR mix (Qiagen).

### MicroRNA extraction and analysis

Extraction of total RNA and microRNA was performed using the QIAsymphony RNA Kit (Qiagen) according to the manufacturer's protocol. In addition, the samples were treated with DNAse to avoid contamination by genomic or mitochondrial DNA. A total of 10 ng of RNA was used for cDNA preparation using the MiScript II RT Kit (Qiagen) and the mmu-miR-122 transcript levels were measured by qPCR on the LightCycler480 (Roche), using the miScript SYBR Green PCR kit with mmu-miR-122 miScript primer assay (Qiagen). According to the supplier's protocol, MicroRNA expression was determined as absolute quantification. To estimate the absolute copy number of miR-122, a standard range was established by 10-fold dilution over 10 points using mus musculus miR-122 (mmu-miR-122) mimic (Dharmacon). The dilution range used for reverse-transcription with miScript Reverse Transcription kit (Qiagen) started from 7.13 10^11^ copies per ng of total RNA for mmu-miR-122 mimic. Copy number of miR-122 per ng of total RNA in each sample was determined by plotting Ct values in the mmu-miR-122 standard curves.

### Cell proliferation assay

Cell proliferation assay was performed on BMOL cells pretreated with 50 ng/mL IL-17 for 20 and 40 days using 3-(4,5-dimethylthiazol-2-yl)-5-(3-carboxymethoxyphenyl)-2-(4-sulfophenyl)-2H-tetrazolium (MTS) purchased from Promega. Two thousand five hundred cells per well were seeded in 200 µL William's medium supplemented with 5% FCS, in 96-well plates in six replicates and incubated for 6 hours, 1, 3, 7 and 8 days. The medium was changed every 2 days without adding recombinant mouse IL-17. After the treatment period, the medium was replaced with 100 µL DMEM without phenol red (Gibco) and 20 µL MTS agent was added to each well. The assay is based on the bio-reduction of MTS by live cells into a colored formazan product that can be quantified by absorbance measurement at 490 nm (Biotek ELX808LBS reader), and optical density (OD) values normalized with control BMOL.

### Protein analysis

Total proteins from BMOL cells cultured for 20 or 40 days in William's medium with 5% FCS with or without IL-17 were extracted with ice-cold RIPA lysis buffer and quantified with the BCA kit (Pierce). Proteins were electrophoresed on SDS-PAGE gels and transferred to a nitrocellulose membrane (Invitrogen) after blocking with 5% BSA. Ten µg of proteins were added to each well. Protein detection was done using the following antibodies: anti-cyclin D, anti-cyclin E, anti-p21 from Santa Cruz Biotechnologies, anti-Retinoblastoma (RB), anti-phosphorylated RB and anti-c-Raf from Cell Signaling Technology and anti-β-actin from Sigma-Aldrich. The membrane was incubated with the secondary antibodies (Horseradish peroxidase) and signals were detected with ECL reagent (GE Healthcare).  The band intensity was measured by using Image J software (NIH).

### *In situ* Proximity Ligation Assay

BMOL cells (20,000 cells) were treated with 50 ng/mL of IL-17 for 40 days and seeded in Lab-Tek chambers overnight at 4 °C. Adherent cells were fixed with 4% PFA in PBS and saturated with 0.1% BSA fraction V at 37 °C for an hour. Cells were incubated with anti-mouse cyclin E, and anti-mouse p21 or with anti-mouse cyclin D and anti-mouse p21 monoclonal antibodies, each at a 1:50 dilution for an hour at 37 °C. Ligation, amplification and detection were performed with Duolink *in situ* Orange Starter Kit. Anti-Mouse/Rabbit were used according to the manufacturer's instructions and images were acquired using a Zeiss Axioskop 40 microscope equipped with Zeiss AxioCAM image capture system.

### IL-17 quantification

Mouse IL-17 Quantikine ELISA kit (R&D, M1700) was used to quantify IL-17 produced by BMOL cells transfected with plasmid pUNO1-mIL17A in culture supernatant before allograft, and in mouse sera after allograft. The procedure was performed according to the protocol described in the manual.

### Transfection of miR-122-mimic

BMOL cells were prepared in 6-well plates for microRNA (miRNA)-mimic transfection. Twenty-five nM of hs-miR122-mimic (Dharmacon) was prepared with DharamaFECT1 reagent (Dharmacon), according to manufacturer's instructions and cells were lysed 48 hours after miR-122 transfection for RNA analysis.

### Small and mRNA library preparation and sequencing

Using the TruSeq Small RNA Sample Prep Kit (Illumina), total RNA, containing small RNAs, was reverse transcribed into a cDNA library. In brief, 1 µg of total RNA per sample was ligated with 5' and 3' adapters, reverse transcribed, treated with RNase, and amplified with PCR using specific labeled amplification primers. To select the size of small RNAs, samples were migrated on 6% native polyacrylamide gels. To collect miRNA populations, cDNA fragments between 145 and 160 bp were cut from the gel, eluted and precipitated. Finally, cDNA chip was dried and suspended in 10 µL of nuclease free water. In parallel, cDNA library was built from total RNA (1 µg per sample) from the same samples. Purification and fragmentation were performed using the TruSeq RNA Sample Preparation Kit v2 - Set A (Illumina). Next, cDNA samples were end repaired using End Repair Mix reagent (Illumina) and double-stranded cDNAs were purified and enriched to create the cDNA library. The quality of cDNA in each final miRNA and mRNA libraries was assessed using the Agilent 4200 TapeStation System (Agilent) and the amount was verified using Quant-iT PicoGreen dsDNA Assay Kit (Invitrogen). Equimolar concentrations of each final library were pooled at a final concentration of 2 nM of cDNA. Libraries were run on the Illumina NextSeq 500 platform using the High Output kit v2 (75 cycles, Illumina).

### Bioinformatics analyses of microRNA data

Quality control of sequence reads generated from Illumina-based next-generation sequencing was assessed using Fastqc (v0.11.5) and trimmed reads (trimmomatic v0.36) were aligned to the mouse reference miRNAs (miRBase v21) of the latest mouse genome assembly by using Bowtie (v2.0.6) to identify known miRNAs. Differences in normalized expression levels of miRNAs in IL-17-treated LPC compared to untreated cells were carried out by the DESeq2 R package (Bioconductor) using a model based on the negative binomial distribution. P-values were adjusted for multiple testing using the Benjamini-Hochberg method for controlling the false discovery rate (FDR). MiRNAs with a corrected p-value < 0.05 and fold changes either >1.5 or <0.67 were assigned as differentially expressed.

### Statistical analysis

Results are expressed as mean ± S.E.M., and statistical significance was determined by a two-tailed Student's t test or Mann and Whitney U test as appropriate using PRISM 5.0 software. Data were considered significantly different for * p < 0.05, ** p < 0.01, *** p < 0.001.

## Results

### The expression of the CSC marker CD133 is correlated with the LPC marker CK19 in human hepatocellular carcinoma

**Figure 1:**
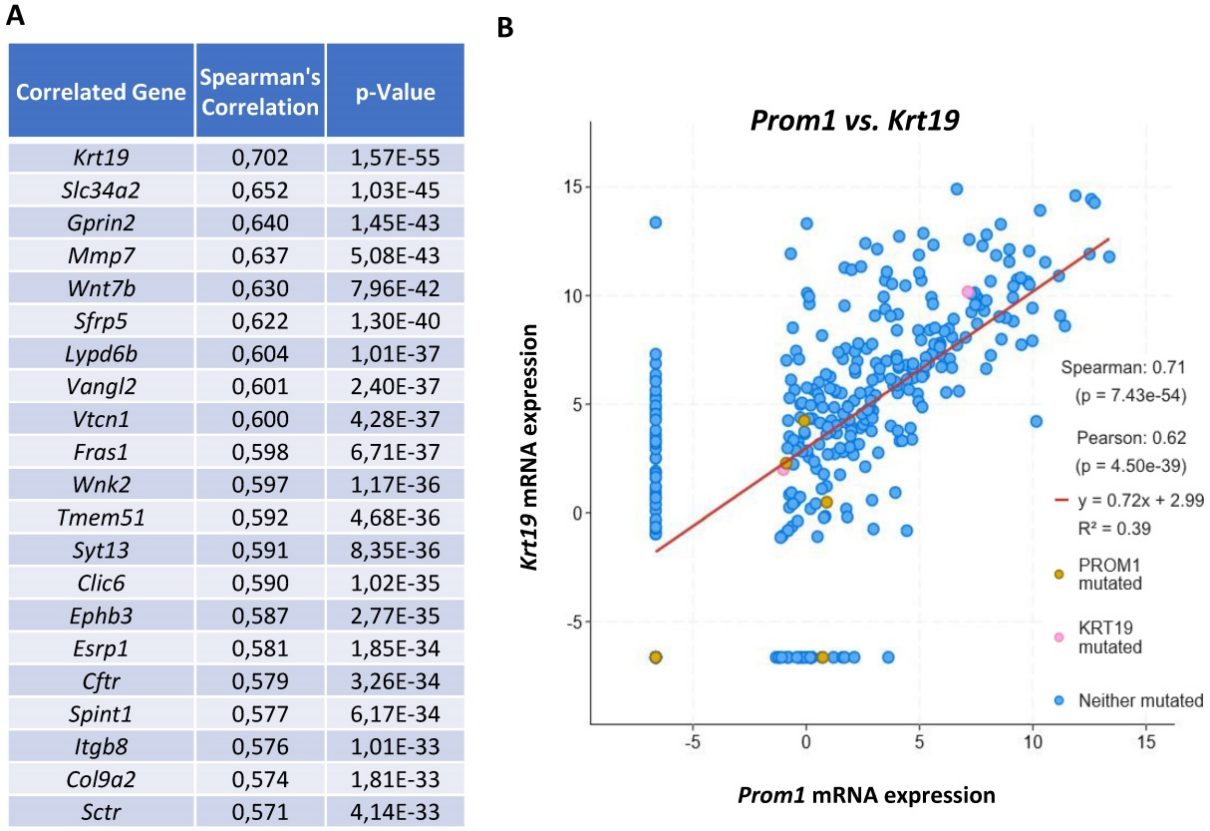
** The expression of the CSC marker CD133 is correlated with the LPC marker CK19 in human hepatocellular carcinoma. (A)** Visualization of top 20 genes that are correlated with *Cd133* known as *Prom1* mRNA expression with the highest correlation with *Ck19* known as *Krt19* (RSEM, batch normalized from Illumina Highseq_RNASeqV2; n=366 HCC samples). **(B)** Correlation between *Prom1* and *Krt19* mRNA expressions (RSEM, batch normalized from Illumina HiSeqV2) represented with Log scaled axis using a linear regression analysis.

In order to determine whether CSCs could originate from normal liver progenitor cells (LPC) and their involvement in liver carcinogenesis, we used the public cBioPortal site for web-based analysis of Liver Hepatocellular Carcinoma (TCGA PanCancer Atlas) and Spearman's correlation coefficient to assess the relationship between the mRNA expression levels of the classically used cancer stem cell marker CD133 (alias PROM1) and other genes 23, 24. The Figure 1 represents a web-based analysis from the Cancer Genome Atlas using 366 HCC samples. The correlation analysis showed that CD133 displayed the highest positive correlation with the expression of CK19 (alias KRT19) which constituted the main marker of LPC/biliary cells (**Figure 1A and B**). This result suggests a possible origin of CSCs from LPCs in human HCC.

### IL-17-producing cells and CSCs are closely located in human cirrhotic livers

**Figure 2:**
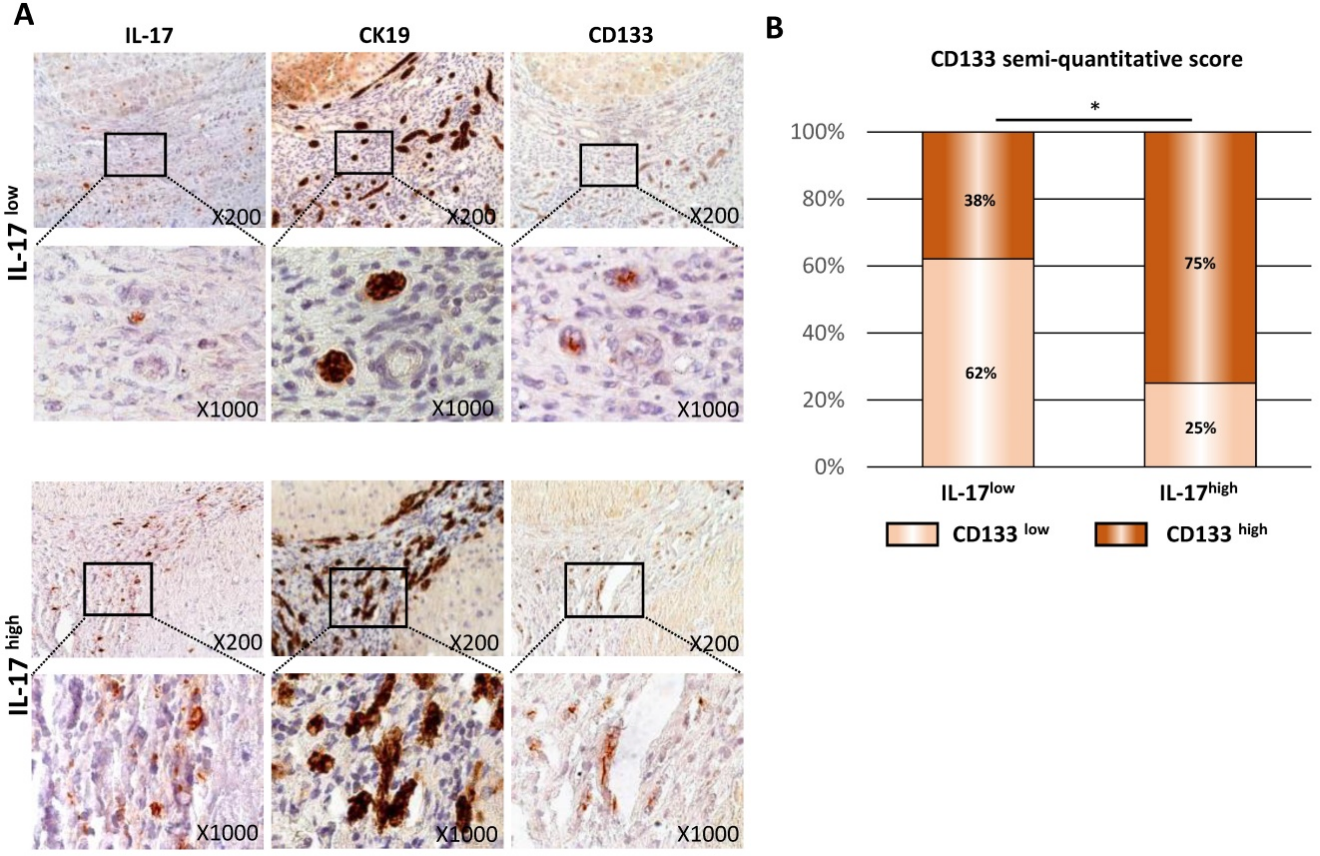
** IL-17-producing cells and CSCs are closely located in human cirrhotic livers. (A)** Interleukin-17A (IL-17), cytokeratin-19 (CK19) and CD133 immunostainings on serial sections of preneoplastic hepatocellular lesions in human livers. **(B)** Relative CD133 immunolabelling was quantified in the two groups of patients with high (n=16) versus low (n=29) IL-17 expression, and statistical differences between the groups were assessed with a Chi-square test.

In human cirrhotic livers, we previously reported a correlation between the number of IL-17-producing cells and the expansion of CK19^+^ biliary and progenitor cells 17. In order to assess whether an increased and sustained IL-17 production contributes to triggering LPC transformation into CSCs, the expression of IL-17, CK19 and CD133 were assessed by immunohistochemistry on serial sections of cirrhotic livers from various etiologies 17 (**Figure 2A**) organized in two groups of patients, based on the number of IL-17^+^ cells infiltrating the liver: IL-17^Low^ and IL-17^High^. In both groups, IL-17-producing cells were located within ductular reactions in close vicinity of CK19^+^ LPCs and CD133^+^ CSCs. In addition, the numbers of CK19^+^ and CD133^+^ cells increased with the number of IL-17-producing cells infiltrating the liver (**Figure 2B**). Semi-quantitative analysis showed a positive correlation between the number of infiltrated IL-17^+^ cells and CSC accumulation. In the group of patients with low levels of IL-17-infiltrating cells defined as IL-17^Low^ patients, 38% of cases were expressing high levels of CD133. Contrastingly, among patients displaying high levels of IL-17-infiltrating cells (IL-17^High^ patients), 75% of cases highly expressed CD133 marker (p<0.05). Collectively, these findings reveal an association between the increased number of IL-17-producing cells and the accumulation of CSCs in human cirrhotic livers.

### Long-term stimulation of LPCs by IL-17 triggers their malignant transformation into a CSC phenotype

#### Long-term stimulation of LPCs by IL-17 enhances their expression of cancer and stemness markers

**Figure 3:**
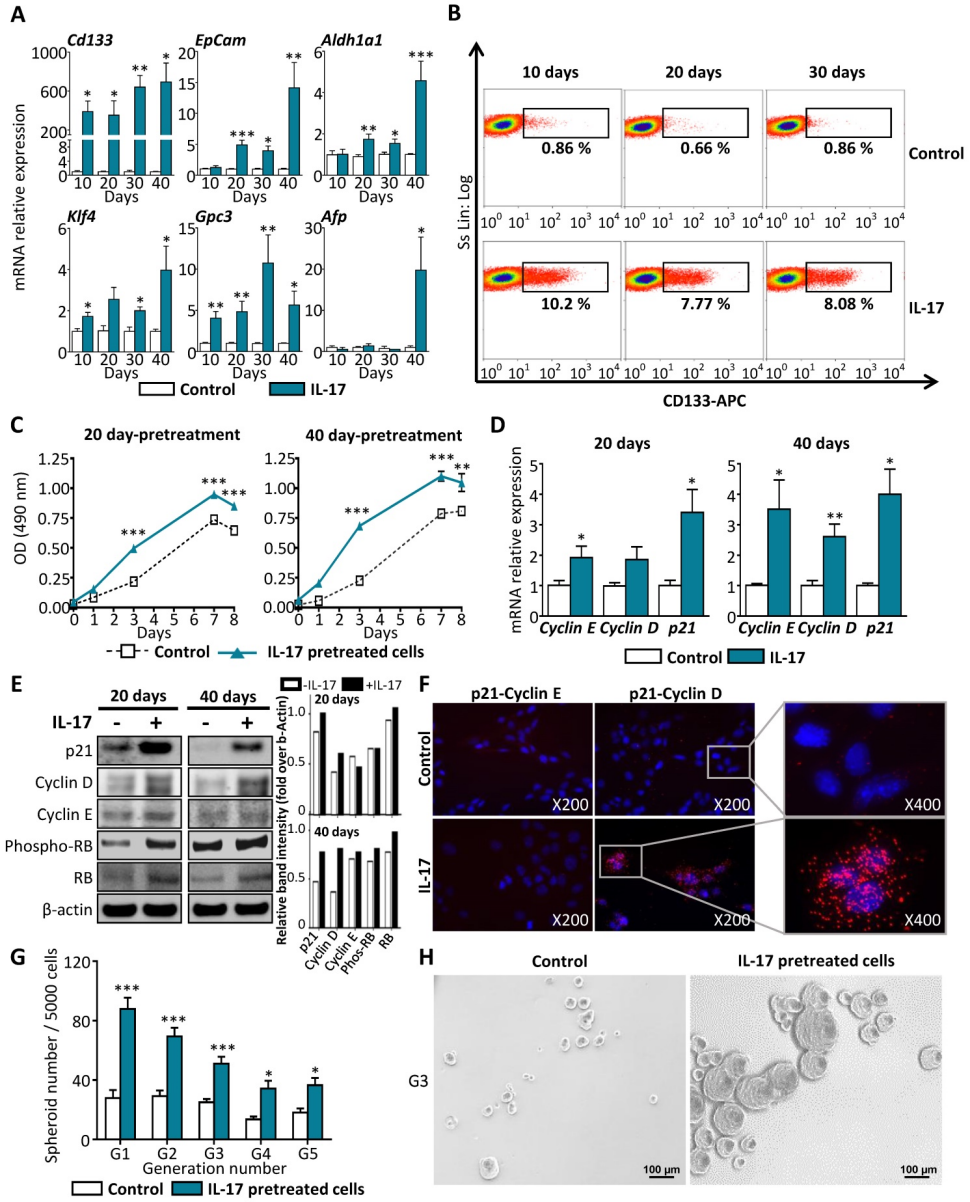
**  Long-term stimulation of LPCs by IL-17 triggers their malignant transformation into a CSC phenotype. (A)** LPC were stimulated for 10 to 40 days with IL-17 and relative mRNA expression of cancer (*Gpc3* and *Afp*) and stem cell markers (*Cd133, Epcam, Aldh, Klf4*) were analyzed by RT-qPCR and expressed as fold change over untreated cells (control). **(B)** CD133-positive LPCs were quantified by flow cytometry analysis after IL-17 treatment for 10 to 30 days. **(C)** After pretreatment with IL-17 for 20 and 40 days, LPC proliferation rate was assessed every day for 8 days using MTS test. Data are representative of 3 independent experiments. **(D)** Fold change in *Cyclin D1*, *Cyclin E* and *P21* mRNA expression was determined by relative qPCR analysis of LPC treated with IL-17 or vehicle (control) for 20 or 40 days. **(E)** Western blot analysis of cell cycle related-proteins p21, cyclin D and, E and total retinoblastoma (RB) or phosphorylated retinoblastoma protein (phospho-RB) in LPCs treated or not (control) with IL-17, for 20 or 40 days. The western blot band intensity is quantified by using image J software. The graphs represent the intensity of each band as ratio over b-actin band intensity. **(F)** Cyclin D and E interaction with p21 was assessed by the Duolink Proximity Ligation Assay on LPC treated with IL-17 for 40 days and shown by the presence of red spot (insets with higher magnification). Nuclei were stained with DAPI. Close-proximity of p21 and cyclin D (red) is only observed in IL-17-treated LPC. **(G)** After 30 days of IL-17 pretreatment or without pretreatment (control), LPC were cultured in sphere formation condition assay over 5 generations and total spheroid number per well (larger than 40 μm) was counted for each generation (from G1 to G5). Experiments were performed in sextuplicate in 3 independent experiments. (H) Representative micrographs show formed spheroids (scale bar 100 μm) at G3 from LPC pretreated (or not, control) with IL-17. Data represent mean ± SEM for 3 independent experiments performed in triplicate and *P < 0.05; **P < 0.01; ***P < 0.001 for control versus IL-17-treated or pretreated cells.

To investigate potential effects of IL-17 on LPC transformation *in vitro*, a non-tumor murine LPC line (BMOL) was cultured for long-term (10, 20, 30 or 40 days) with or without IL-17. In those conditions, mRNA expression of CSC (*Cd133, Epcam* and *Aldh*), of pluripotency (*Klf4*) and of tumor cell (*Gpc3* and *Afp*) markers were found significantly induced by IL-17 when compared to those from control non-treated LPCs (**Figure 3A**). Furthermore, flow cytometry analysis showed that 8 to 10% of IL-17-treated LPCs acquired CD133 protein expression after 10, 20 or 30 days but not in non-treated LPCs (**Figure 3B**). Similar neo-expression of CSC-related genes induced by IL-17 was evidenced in human hepatic progenitor cell line HepaRG (**Supplemental Figure 1**). In this model, sustained IL‐17 treatment reduced the mRNA expression of the two hepatocytic markers *Alb* and *Hnf4α* (**Supplemental Figure 1A**), while inducing mRNA expression of stem cell markers such as *Cd133* and *Epcam* (**Supplemental Figure 1B**). These results strongly suggest that IL-17 promotes the transformation of both murine and human LPCs into a CSC phenotype.

#### Long-term stimulation of LPCs by IL-17 enhances their self-renewal properties

In a next step, we proposed to determine whether the acquisition of IL-17-induced CSC marker expression by LPCs was associated with enhanced cell cycle activity and self-renewal capacities. For that purpose, an MTS cell proliferation assay was performed on BMOL cells pretreated with IL-17 for 20 or 40 days. Then, the proliferation rate was measured every day for 8 days of culture without adding IL-17 in the medium. Under these conditions, IL-17-pretreated cells for either 20 or 40 days, had acquired enhanced cell cycle activity when compared to non-pretreated control cells (**Figure 3C**). This effect was associated with a significant increase in *Cyclin D1*, *Cyclin E* and *p21 (CDKN1A/waf1)* at mRNA (**Figure 3D**) and at protein levels (**Figure 3E**) in BMOL cells pretreated with IL-17 for 20 or 40 days. The protein levels quantified by image J showed an increase in P21, Cyclin D, E and Retinoblastoma expression at 20 and 40 days under IL-17 stimulation (**Figure 3E**). Depending on which partner p21 is interacting with, its impact on cell cycle regulation will be different. The interaction of p21 with Cyclin E prevents cell-cycle progression by inhibiting the activity of cyclin E/CDK2 complexes. In contrast, p21 interaction with Cyclin D1 results in retinoblastoma (RB) hyperphosphorylation and activation of mitogenic pathway 25. To identify p21 partner in LPCs treated with IL-17, a PCR Ligation-Assay (PLA) was performed. A close proximity of p21 and cyclin D was evidenced by increased fluorescent dots in condition where LPCs are treated with IL-17 and no interaction between p21 and cyclin E was observed (**Figure 3F**).  Furthermore, a 5-generation spheroid formation assay was performed *in vitro* as a functional test assessing the self-renewal capacity of these cells. Only spheroids with diameters higher than 40 µm were counted. We showed that LPCs pretreated with IL-17 for 30 days had acquired self-renewal properties as compared to non-pretreated cells from the first generation (**Figure 3G**).  This effect was maintained for 5 generations and illustrated by a microscopic photography taken at the third generation (**Figure 3H**). These results indicate that IL-17 pretreatment enhances LPCs self-renewal capacity, and this effect is maintained over generations. Taken together, these findings demonstrated that long-lasting IL-17 pretreatment of LPCs leads to their conversion into cells with a CSC phenotype.

### IL-17 induces LPC transformation into CSCs through miR-122 down-regulation

**Figure 4:**
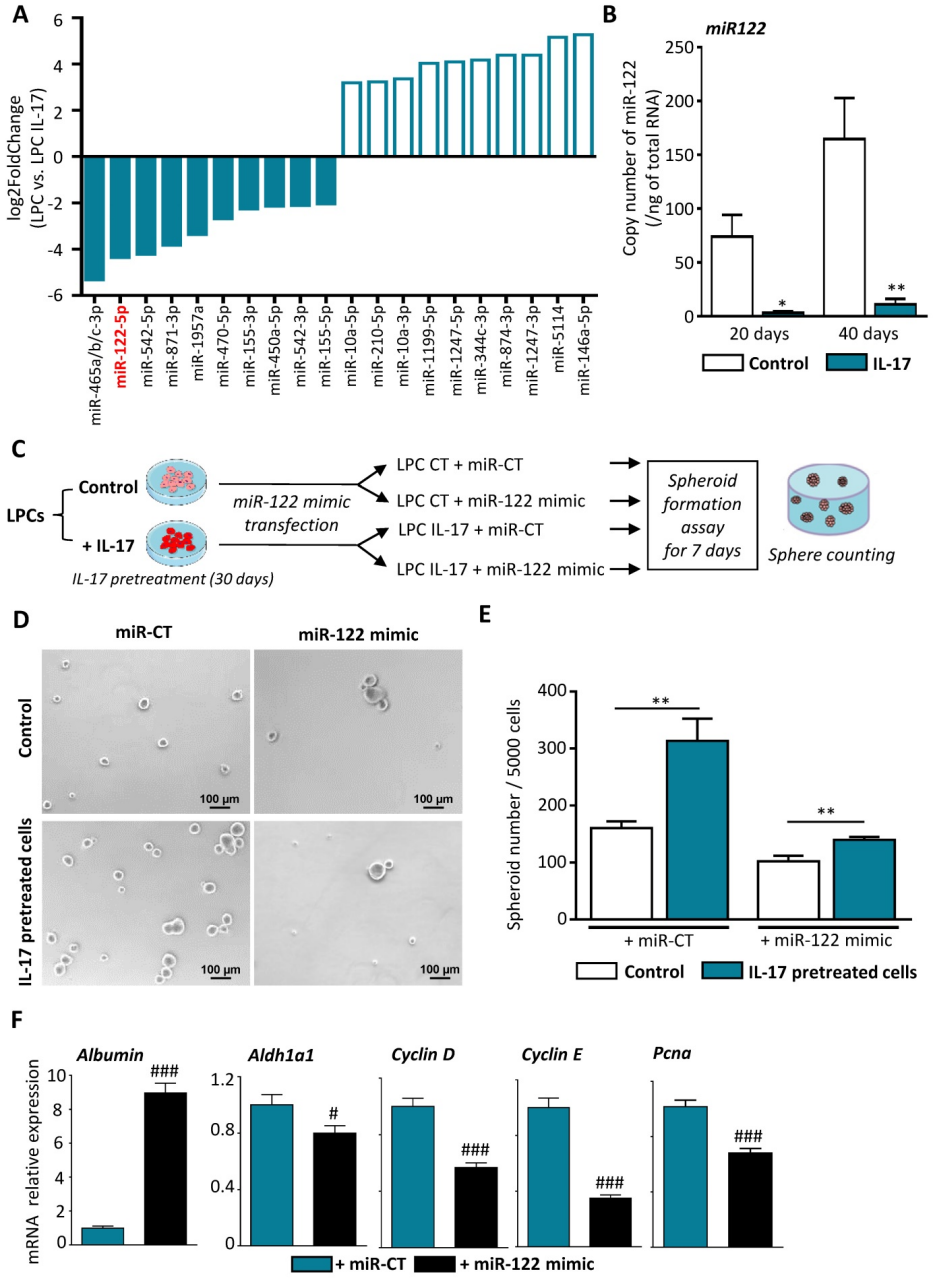
** IL-17 induces LPC transformation into CSCs through miR-122 downregulation. (A)** Top 10 up (white bars) and down (blue bars) of the most dysregulated microRNAs in IL-17-treated LPC compared to untreated cells after 40 days of culture. **(B)** Expression of miR-122 was assessed by qRT-PCR in IL-17-treated or not (control) LPC for 20 or 40 days. **(C)** Control (LPC CT) or IL-17-pretreated LPC for 30 days (LPC IL-17) were transfected with either a control miRNA (mir-CT) or miR-122 mimic and **(D)** next cultured in sphere formation assay for 7 days (scale bar 100 μm). **(E)** Representative micrographs showed number of formed spheroids with a diameter greater than 50 µm **(F)** Messenger RNA expressions of *Albumin*, *Aldh1A1* and cell-cycle markers (*Cyclin D*, *E*, *Pcna*) were assessed by qRT-PCR in IL-17 pretreated LPC for 30 days and transfected either with miR-CT or miR-122 mimic. Data represent mean ± SEM, n=6 per condition. *P < 0.05; **P < 0.01 for control versus IL-17 treated or pretreated cells; #P<0.05 and ###P < 0.001 for miR-CT treated cells versus miR-122 mimic-treated cells.

Several pieces of evidence suggest that miRNA have regulatory functions in cancer initiation, and dysregulation of miRNAs occurs frequently in a variety of liver diseases, including HCC 26. To determine the underlying mechanism by which IL-17 favors the expansion of CSCs and subsequent tumor initiation and progression, we attempted to identify miRNAs that are dysregulated by IL-17 and could represent a molecular signature of IL-17 effects during hepatocarcinogenesis (**Figure 4A**). Among the most dysregulated miRNAs, miRNome analysis revealed a decrease in miR-122-5p expression in IL-17-treated LPCs when compared to non-treated LPCs. MiR-122-5p is by far the most abundant miRNA expressed in murine and human hepatocytes that has been shown to be downregulated during hepatocarcinogenesis 27, while miR-465a/b/c-3p expression was found only in murine cells. This finding was validated by RT-qPCR showing a 90% decrease of miR-122-5p expression in IL-17 treated cells when compared to untreated LPCs (**Figure 4B**).  To further determine whether miR-122 downregulation could be responsible for LPC transformation, we then transfected LPCs with mir-122 mimic *in vitro* (**Figure 4C**). Efficiency of transfection was validated by qPCR showing a strong elevation of miR-122 expression in both IL-17-treated or not (control) transfected-LPCs (**Supplemental Figure 2**). Overexpression of miR-122 in LPCs abolished self-renewal capacity acquired by IL-17 pretreatment. The number of spheroids obtained from IL-17-pretreated LPCs was higher than the spheroid number formed from untreated LPCs. However, overexpression of miR-122 mimic was sufficient to abolish IL-17-induced self-renewal capacities (**Figure 4D and E**).  In addition, miR-122 mimic overexpression restored albumin expression while reducing the expression of stemness markers such as *Aldh1a1* and of cell cycle related-genes including *Cyclin D, E* and *Pcna* (**Figure 4F**).

### Constitutive expression of IL-17 in LPCs enhances tumor expansion with an aggressive phenotype* in vivo*

**Figure 5:**
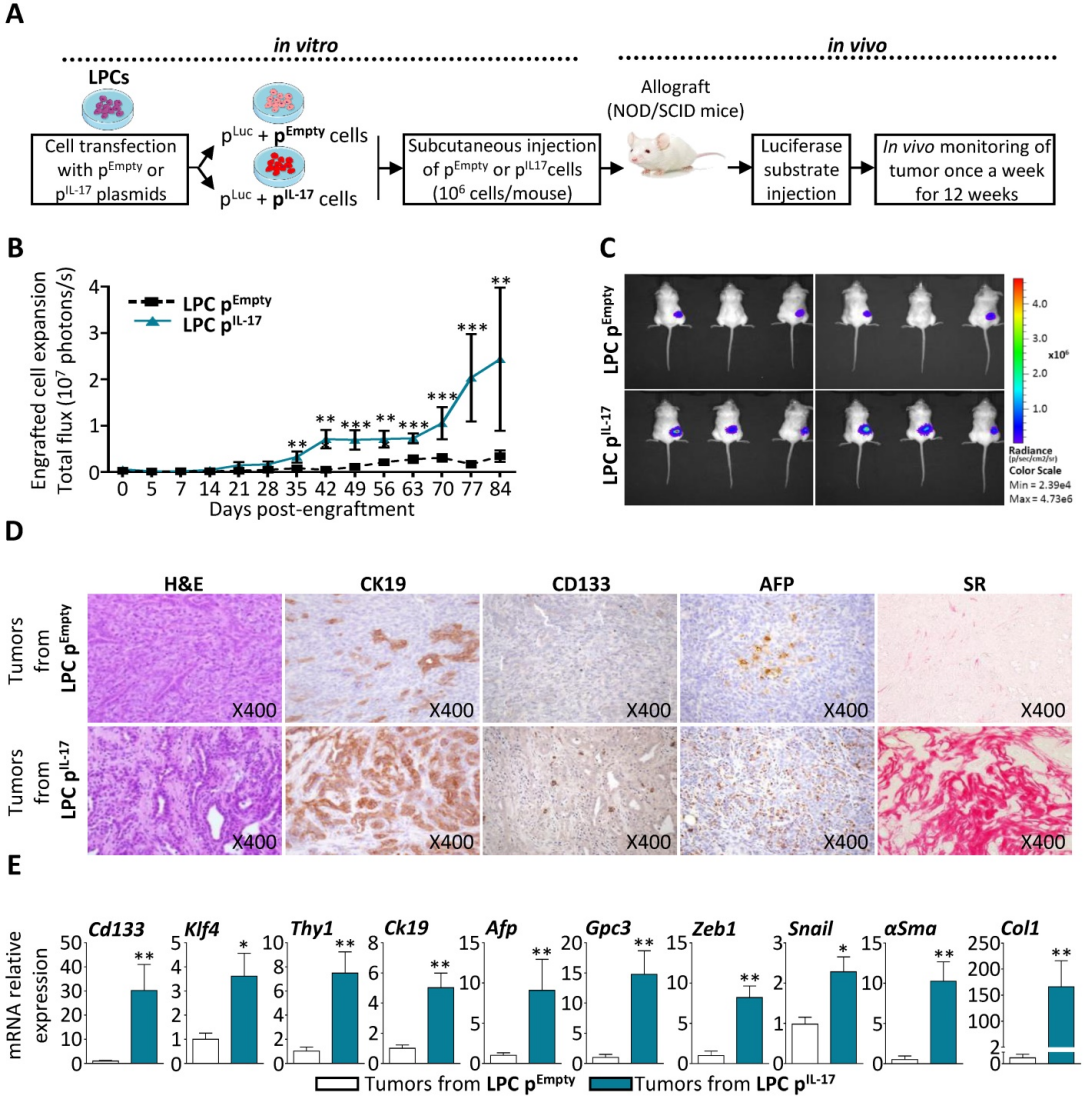
** Constitutive expression of IL-17 in LPCs enhances tumor expansion with an aggressive phenotype *in vivo*. (A)** NOD/SCID mice were inoculated subcutaneously with IL-17/Luc-expressing LPC (LPC p^IL-17^ or Luc-expressing LPC with empty vector (LPC p^Empty^) as controls. **(B)** Engrafted-cell expansion was followed once a week for 84 days by *in vivo* bioluminescence imaging.  **(C)** Representative average radiance (photons/s per square centimeter per steradian) of tumor bearing mice 56 days after cell engraftment according to the associated color scale. **(D)** Histological and immuno-histochemical analyses of tumors 84 days after LPC inoculation was performed. **(E)** Messenger RNA expression of CSC (CD133, KLF4, Thy-1, CK-19, AFP and GPC3), and EMT/fibrotic (Zeb-1, Snail, α-SMA and Col-1) markers were assessed by RT-qPCR in isolated tumors at 84 days. Data represent mean ± SEM, n= 6 mice per group and *P < 0.05; **P < 0.01; ***P < 0.001 for control LPC p^Empty^ versus p^il-17^ engrafted mice.

To further characterize IL-17 tumorigenic effect *in vivo*, an LPC line highly expressing IL-17 was generated using a stable co-transfection with mouse IL-17 plasmid (LPC p^IL17^) or an empty vector as control (LPC p^Empty^), combined with pGL4.51 (Luc2) vector in BMOL cells. Transfected cell phenotype was verified prior to *in vivo* engraftment and the results showed that IL-17 constitutive expression up-regulated cancer cell and CSC markers (*Gpc3* and *Cd133*) as compared to control cells (**Supplemental Figure 3A, B**). In addition, IL-17 production by transfected-LPCs was verified in the culture medium by ELISA before engraftment (**Supplemental Figure 3C**). Next, LPC p^IL17^ or LPC p^Empty^ cells were subcutaneously engrafted into immunodeficient NOD/SCID mice. Their expansion was subsequently monitored for 12 weeks by bioluminescence imaging (**Figure 5A**). Engrafted LPC p^IL17^ cells significantly expanded, whereas LPC p^Empty^ cells did not (**Figure 5B**). A representative figure illustrates the higher-level of bioluminescence reflecting the number of expanded cells, recorded at day 84 in mice engrafted with LPC p^IL17^ when compared to those engrafted with LPC p^Empty^ cells (**Figure 5C**). Histological analysis of the tumors after an H&E staining revealed a typical morphological aspect of mixed HCC and cholangiocarcinoma in IL-17-enriched tumors, but not in tissues obtained from LPC p^Empty^ -engrafted cells (**Figure 5D**). The tumor phenotype obtained from LPC p^IL17^ -engrafted cells was associated with a greater expression of the CK19 biliary/progenitor cell marker, and an increased number of CD133^+^ and AFP^+^ cells. This was also associated with increased fibrosis as revealed by Sirius red (SR) staining.

In order to characterize the tumors deriving from LPC p^IL17^ cells, an extraction of RNA from tumor tissues from both engrafted mice was performed. The obtained results showed that tumors from LPC p^IL-17^ have a significant increase in cancer cell and CSC markers (*Cd133, Klf4, Thy1, Ck19, Afp* and *Gpc3*) when compared to tumors from LPC p^Empty^ (**Figure 5E**). A significantly increased expression of Epithelial-Mesenchymal Transition (EMT)-related genes (e.g. *Snail* and *Zeb1*) and of fibrosis-related genes (e.g. *αSma* and *Col1*) was also observed in LPC p^IL17^-derived tumors. Collectively, these data revealed that an IL-17-enriched microenvironment confers an aggressive phenotype to LPC-derived tumors with histopathological features of mixed HCC and cholangiocarcinoma phenotype.

### Constitutive production of IL-17 triggers stem-like phenotype transformation of LPCs, initiates liver fibrogenesis and downregulates hepatic miR-122 expression

**Figure 6:**
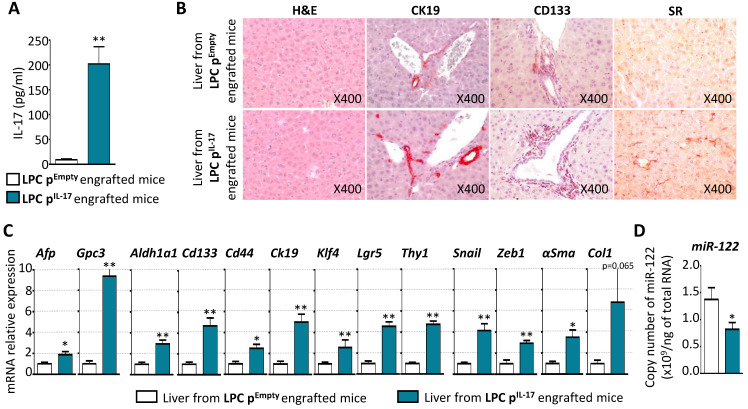
** Constitutive production of IL-17 trigger stem-like phenotype transformation of LPCs, initiates liver fibrogenesis and downregulates hepatic miR-122 expression. (A)** Plasma concentrations of IL-17 in LPC p^Empty^ or LPC p^IL-17^ engrafted NOD-SCID mice. **(B)** Representative microphotographs of Hematoxylin-Eosin, CK19, CD133, and Sirius Red (SR) stainings in the liver from LPC p^Empty^ (upper panel) or LPC p^IL-17^ (lower panel) engrafted mice (X original magnification). **(C)** mRNA relative expression analysis of tumor (*Afp, Gpc3*), CSC (*Aldh1a1, Cd133, Cd44, Klf4, Lgr5, Thy1*), fibrotic (*αSma, Ck19, Col1*) and EMT (*Snail, Zeb1*) markers by qPCR in the liver from LPC p^Empty^ or LPC p^IL-17^ engrafted mice. *P<0.05, **P<0.01: liver from LPC p^Empty^ vs LPC p^IL-17^ engrafted mice. **(D)** Total copy number of miR-122 per ng of total RNA assessed by qPCR in the liver from LPC p^Empty^ or LPC p^IL-17^ engrafted mice. *P < 0.05; **P < 0.01: liver from LPC p^Empty^ vs LPC p^IL-17^ engrafted mice. Data represent mean ± SEM, n= 6 mice per group.

To determine the impact of systemic IL-17 on liver, serum IL-17 levels in LPC p^Empty^ and in LPC p^IL-17^ engrafted-mice were measured 12 weeks after allograft (**Figure 6A**). Serum IL-17 levels reached 202±34 pg/mL, a range comparable to that observed in cirrhotic and HCC patients 28. Histological examination of livers from LPC p^Empty^ or p^IL-17^ engrafted mice after 12 weeks, did not reveal any difference in hepatocyte morphology or liver architecture (**Figure 6B**). In contrast, constitutive IL-17 release in the blood increased hepatic CK19 and CD133 immunostaining, and sinusoidal fibrogenesis as revealed by Sirius Red (SR) staining, in the livers from LPC p^IL-17^-engrafted mice (**Figure 6B**). Moreover**,** RT-qPCR analysis showed enhanced expression of HCC (*Afp, Gpc3*), CSC (*Aldh1a1, Cd133, Cd44, Klf4, Lgr5, Thy1*), EMT (*Snail, Zeb1*) and fibrosis (*αSma*) related-genes in the livers from LPC p^IL-17^-engrafted mice (**Figure 6C**). In order to determine whether IL-17 impacts miR-122 expression in the liver, an extraction of total RNAs including small RNAs from livers of LPC p^Empty^ or p^IL-17^-engrafted mice was performed. Interestingly, analysis of miR-122 expression by qPCR revealed that IL-17 sustained production significantly decreased miR-122 expression in livers from LPC p^IL-17^-engrafted mice compared to LPC p^Empty^-engrafted animals (**Figure 6D**). Together, these results indicate that long-term systemic IL-17 production induces stem-like phenotype transformation of LPCs, favors liver fibrogenesis and downregulates hepatic miR-122 expression.

### IL-17-neutralizing strategies reduce tumor growth by limiting CSC occurrence and preventing miR-122 downregulation *in vivo*

**Figure 7:**
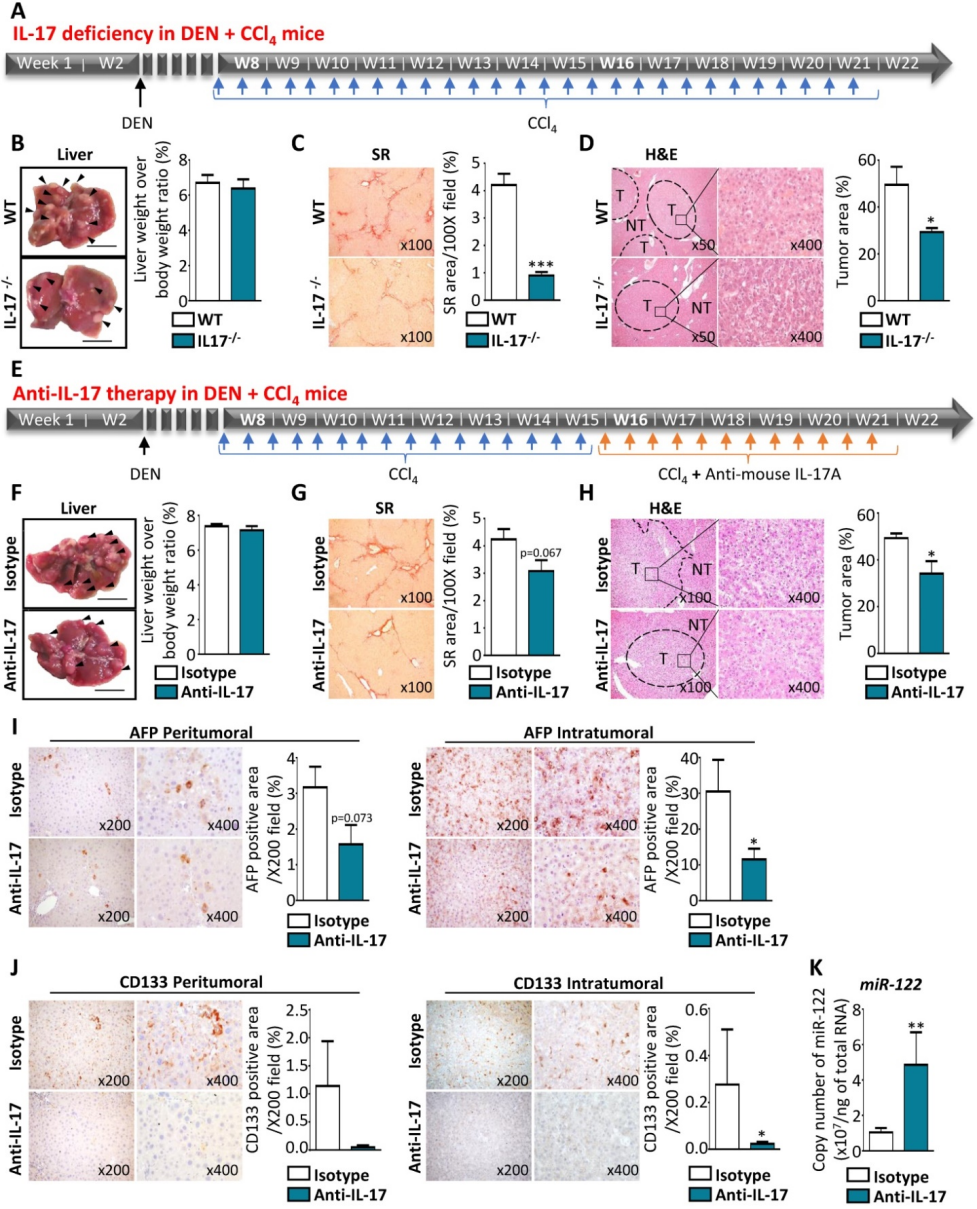
** IL-17-neutralizing strategies reduce tumor growth by limiting CSC occurrence and preventing miR-122 downregulation *in vivo*. (A)** Schematic representation of experimental design used for chronic DEN and CCl_4_ administration in WT or IL-17^-/-^ mice. **(B)** Representative macroscopic liver morphology from 22-week-old WT or IL-17^-/-^ mice subjected to the DEN and CCl_4_ murine model of liver cancer (left panel with scale bars represent 1 mm and arrows show tumor nodules). Quantification of liver weight over body weight ratios (right panel). **(C)** Representative microphotographs and quantification of Sirius red staining in the liver of WT and IL-17^-/-^ mice. **(D)** Hematoxylin-Eosin staining allows to highlight non-tumor (NT) and tumor (T) liver tissues at two different magnifications (left x500 and right x400) and quantification of tumor areas in the liver of WT and invalidated mice (right). **(E)** Experimental design for anti-IL-17 therapy in DEN+ CCl_4_-induced HCC model. **(F)** Representative macroscopic liver morphology (scale bar 1 mm and arrows show tumor nodules and liver weight over body weight ratios measure in mice treated by control isotype or anti-IL-17 (right). **(G)** Sirius red staining (left) and tissue collagen quantification (right) in the liver from mice treated by control isotype or anti-IL-17 antibodies. **(H)** Representative microphotographs of Hematoxylin-Eosin staining showing non-tumor (NT) and tumor (T) areas, and quantification in mice treated with control isotype or anti-IL-17 antibodies (right).** (I)** Representative immunohistochemistry staining for AFP and signal quantification in peritumoral or intratumoral areas. **(J)** Representative CD133 immunostaining and signal quantification in peritumoral and intratumoral areas. **(K)** Absolute quantification of miR-122 by qPCR. Data represent mean ± SEM and *P<0.05 **P<0.01 mice treated with anti-IL17 (n=7) versus isotype control (n=6) antibody.

To understand how IL-17 impacts tumorigenesis in the liver, tumor formation was measured *in vivo* using a murine model of HCC induced in a fibrotic context by DEN+CCl_4_ administration (**Figure 7A**). Macroscopic analysis of livers from DEN+CCl_4_ mice revealed that the number of nodules was lower in the livers of IL-17-deficient mice treated with DEN+CCl_4_, as compared to wild-type (WT) treated-mice (**Figure 7B, left panel**), whereas the liver weight to body weight ratio was similar in the two groups (**Figure 7B, right panel**). Furthermore, IL-17-deficient mice displayed a significant reduction of hepatic fibrosis (**Figure 7C**) and tumor areas (**Figure 7D**). Collectively, these data suggest that IL-17 blockade could reduce the tumor burden *in vivo.* To verify this hypothesis, WT mice treated with DEN+CCl_4_ were intraperitoneally injected twice a week with 100 µg of anti-IL-17 neutralizing antibody or control isotype and for 6 weeks (**Figure 7E**). On macroscopic examination, the livers of anti-IL-17-treated DEN+CCl_4_ mice harbored fewer multi-subcapsular nodules of less than 5 mm than those from isotype control-treated mice, whereas similar liver weight to body weight ratios and fibrosis levels were observed in the 2 groups (**Figure 7F and G**). Tumor area quantification using the QuPath software on whole digital H&E slides showed a significant reduction of the percentage of tumor area in the anti-IL-17-treated mice group, as compared to the control group (**Figure 7H**). Moreover, IL-17 blockade led to a 2-fold decrease in AFP^+^ (**Figure 7I**) and CD133^+^ (**Figure 7J**) cells counted in both peri- and intra-tumor areas. Finally, RT-qPCR analysis revealed a strong elevation of miR-122 expression in tumor (**Figure 7K**) and non-tumor parenchyma (data not shown) from DEN+CCl_4_ mice that received anti-IL-17 therapy. Together, these results demonstrated that IL-17-deficiency or anti-IL-17 therapy led to reduce liver tumor development and allowed to inhibit LPC transformation into CSCs, most likely by restoring miR-122 expression.

## Discussion

The cellular origins and the molecular events that drive primary liver cancer occurrence and growth remain unclear. Recent findings suggest that CSCs can play the role of tumor-initiating cells 6. One of the questions that needs to be addressed is whether tumors arise from dedifferentiation of epithelial cells (hepatocytes and biliary cells) or from a blockade of LPC differentiation and cell-cycle dysregulations leading to their transformation into CSCs 5.

Clinical observations in patients with chronic liver diseases revealed an increased number of CK19^+^ LPCs with the severity of the diseases 7, 17 Moreover the presence of stemness markers such as CD133 was associated with a poor prognosis and an aggressive tumor behavior of primary liver cancers 29. While CD133^+^ CSCs were found expressed in up to 40% of HCC 30, they were identified in the majority of ICC 31, and their presence was associated with poor clinical outcomes.

In experimental animal models, LPCs have been described as stem/progenitor cells contributing to liver carcinogenesis 4. Remarkably, subcutaneous engraftment of LPCs isolated from cirrhotic or HCC into immunodeficient mice led to tumor development in contrast to LPCs isolated from non-cirrhotic or non-cancerous livers 13, 32. Together these findings strongly suggest that LPC transformation into CSCs contributes to the initiation of primary liver cancers. Thus, it is of interest to identify the mechanisms allowing LPC conversion into CSCs 33 to propose suitable strategies preventing cancer initiation and expansion 8.

Primary liver cancers generally develop in a context of chronic inflammation. In that inflammatory context, a recent meta-analysis revealed that IL-17 is associated with a poor prognosis of gastrointestinal tumors 34 and that IL-17 is also considered as a tumor-promoting cytokine that regulates macrophage activation and cholesterol synthesis in hepatocytes in an experimental model of alcohol-induced HCC 35. In addition to a key role described for this cytokine in tumor progression and in resistance to anticancer therapies, the fact that IL-17 is also produced at preneoplastic stages of chronic inflammatory liver diseases suggests its involvement in tumor initiation. Interestingly, IL-17-mediated activation of stem/progenitor compartment has been largely described in gastrointestinal 36, 37, skin 38, ovarian 39, pancreatic 40, breast 41 and prostate 42 cancers. Thus, this proinflammatory cytokine could directly act on the stem cell compartment and thereby contribute to the development of a wide range of malignancies 16. In this study, we showed that IL-17 producing cells are located in close vicinity of CD133^+^ cells and CK19^+^ LPCs in human cirrhotic livers. We previously demonstrated that IL-17 promotes CK19^+^ LPC accumulation within ductular reaction 17 and here, we evidenced a positive correlation between the number of infiltrated IL-17^+^ cells and CSC accumulation in preneoplastic human liver areas. Because CD133 is the main surface marker of CSC populations in various solid tumor types, these results prompted us to hypothesize that in chronic liver diseases, IL-17 could be involved in CD133^+^ cell accumulation as a consequence of a transformation of LPCs into CSCs.

Although the role of various cytokines in cancer initiation has been largely described, their ability to transform normal cells has not been clearly demonstrated yet. In fact, cytokines exert their potential mutagenic effect by favoring the production of highly reactive molecules containing oxygen/nitrogen that can directly damage DNA in hepatocytes 14. Considering TNF-α and IL-6 as the main cytokines secreted by Kupffer cells (the resident macrophages in the liver) that control both hepatocyte proliferation and LPC expansion, the effect of these proinflammatory factors on the malignant transformation of LPCs into CSCs has been evaluated. Long‐term treatment with TNF‐α, but not IL‐6, induced transformation of WB‐F344 rat LPC which exhibited potent tumorigenicity in NOD/SCID mice 13. Interestingly, this chronic TNF‐α exposure triggered aberrant expression of Ubiquitin D and Checkpoint Kinase 2, key mediators of mitosis that cause significant chromosomal instability. Similarly, in our study, we demonstrated both *in vitro* and *in vivo* that long-term IL-17 treatment promotes LPC transformation into CSCs with enhanced self-renewal capacity. Moreover, histological analysis of tumors originating from LPC and growing in IL-17 rich microenvironment revealed an acquired aggressive phenotype (**Figure 6B**). These results are consistent with recent studies showing similar effect of IL-17 on self-renewal capacity of CSCs in different type of cancers 39, 40, 42.

Chronic nonresolving inflammation and tissue damage participate not only to the activation of LPC facultative compartment, but also to their transformation into CSCs 5, 9, 13. Sustained IL-17-dependent inflammation favors pro-inflammatory response in almost all tissues 15, especially in injured liver tissue, in which IL-17 drives hepatic fibrosis and promotes LPC expansion 17 and may also contribute to tumor initiation, progression and metastasis 16. Anti-inflammatory treatment would decrease the onset of HCC. Interestingly, patient having aspirin as a chronic treatment have shown lower risk of developing HCC 43.

Tumor-associated inflammation can lead to epigenetic alterations 26 and the dysregulated expression of miRNAs are functionally linked to CSC development and several types of cancer including liver cancer 44. In this study, miRNome analysis identified dysregulated miRNAs which may be involved in IL-17-triggered transformation of LPCs into CSCs. We observed a sharp decrease of miR-122-5p expression in LPCs after long-term treatment with IL-17 *in vitro*. Interestingly miR-122, which represents approximately 70% of total hepatic miRNAs, plays a pivotal role in cholesterol metabolism and iron homeostasis. Its expression is frequently reduced or abolished in HCC, while miR-122 is considered as a tumor suppressor in the liver 27. In addition, we have shown that miR-122 mimic transfection into LPCs was sufficient to abolish self-renewal capacities acquired after long-term-IL-17 pretreatment. This result is in accordance with a study showing that miR-122 expression is decreased in CSC/CD133^+^ cells from several hepatic cancer cell lines, and that miR-122 re-expression is associated with reduced self-renewal capacities 45. Together our findings and those from the literature suggest that IL-17 can increase LPC self-renewal capacities by negatively regulating miR-122 expression. This could support the idea that miR-122 is an interesting target for cancer therapy and/or prevention by limiting CSC occurrence and their expansion 46, 47. However, taking into account that miR-122 promotes HCV replication 48, restoring miR-122 expression could not constitute a promising therapeutic strategy since miR-122 mimics could enhance hepatitis C liver damage progression.

The underlying mechanisms of cytokine-mediated epigenetic alterations that drive the conversion of LPCs into CSCs are not fully understood. Our previous study clearly revealed that BMOL cell treatment with IL-17 increased their proliferation and reduced mRNA and protein expression of HNF4α, the master regulator of hepatic differentiation. We also demonstrated that IL-17 treatment abolished hepatocyte nuclear factor 4 alpha (HNF4α) expression in freshly isolated LPC from choline-deficient and ethionine-supplemented diet fed mice. There are three HNF4α binding sites present in miR-122 promoter, which may explain how IL-17 decreases miR-122 expression through the suppression of the liver-specific HNF4α 49, 50. Interestingly, we confirmed that long term exposure of human LPC HepaRG to IL-17 also decreased HNF4α expression (**Supplemental Figure 1**). IL-17-mediated reduction of miR-122 levels could also result from signal transducer and activator of transcription 3 (STAT3) activation. Indeed, IL-17 induces IL-6 production that in turn activates STAT3-dependant pathways, thereby promoting the proliferation of HepG2 cells *in vitro* and tumor growth after orthotopic liver transplantation 51. Interestingly, the activation of a complex STAT3-HNF4 inflammatory loop has been evidenced in persistent HCV-infected HCC cell line that was associated with decreased transcription of miR-122 52. Together, these results support the fact that persistent IL-17-mediated STAT3 activation could induce a decrease of HNF4α expression and subsequent silencing of liver-specific miR-122.

**Figure 8:**
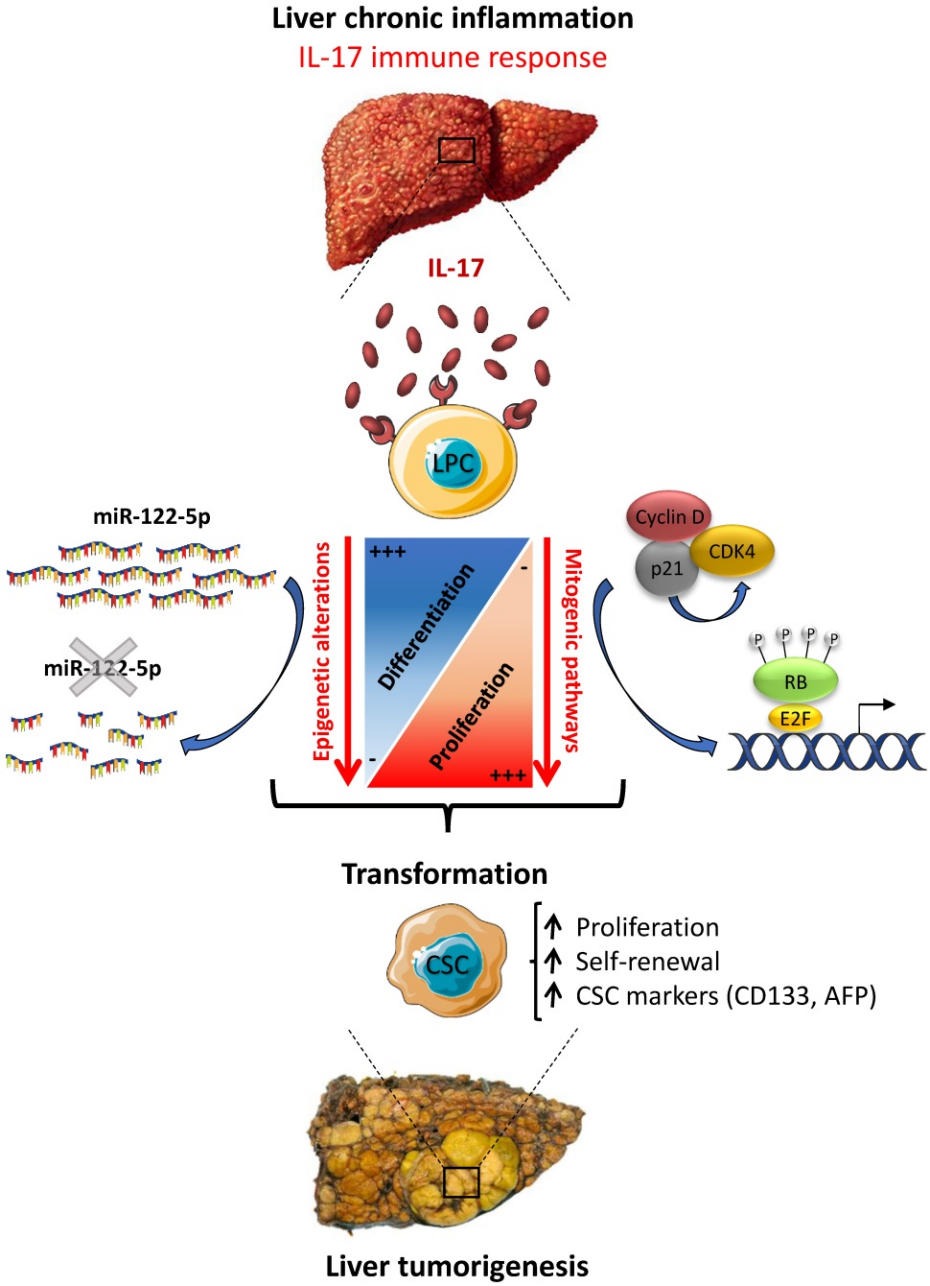
** IL-17 triggers liver cancer development by favoring LPC malignant transformation into CSCs.** IL-17 induces i) LPC de-differentiation by downregulating miR-122 expression and ii) enhances their proliferative and self-renewal capacities by increasing the expression of cell-cycle regulating factors. Altogether, these two pathways lead to LPC transformation into CSCs allowing them to trigger liver cancer development *in vivo*.

In summary, the present work brings novel insights into the mechanisms of liver carcinogenesis, by showing an increased number of infiltrating IL-17-producing cells in preneoplastic cirrhotic livers that correlates with a transformation of LPCs into CSCs. Thus, a sustained IL-17 production might precede or accompany tumor development during chronic liver diseases. Our *in vitro* studies clearly showed that long-term stimulation of LPCs with IL-17 induces their transformation into liver CSCs. In this study, we evidenced that IL-17 promotes LPC stem-cell features and conversely attenuates their hepatocytic differentiation, by promoting mitogenic pathways and de-differentiation through miR-122 downregulation (**Figure 8**).  As a conclusion, our study sheds light on how IL-17 cytokine could initiate primary liver cancer by transforming normal LPCs into tumor initiating cells through epigenetic reprogramming. Taken together, the data suggest that strategies aiming at neutralizing IL-17 production and/or activity, that maintains miR-122 expression in the liver, should be assessed in clinical trials in patients with chronic liver diseases in order to prevent liver cancer initiation from LPC origin, including HCC, ICC and ICC-HCC mixed tumors.

## Supplementary Material

Supplementary figures.

## Abbreviations

BMOL: Bipotential murine oval liver cell
CCl_4_: Carbon tetrachloride
CK19: Cytokeratin 19
CSC: Cancer Stem cell
DEN: Diethylnitrosamine
DR: Ductular reaction
HCC: Hepatocellular carcinoma
ICC: Intrahepatic cholangiocarcinoma
IL-17: Interleukin-17A
LPC: Liver progenitor cell
SR: Sirius red
