# Supplementary material for: Interleukin-17 programs liver progenitor cell transformation into cancer stem cells through miR-122 downregulation with increased risk of primary liver cancer initiation
Source: Int J Biol Sci. 2022 Feb 21;18(5):1944–60. doi: 10.7150/ijbs.70408 (PMC8935237; doi:10.7150/ijbs.70408)

**Supplemental Figure 1:** Long-term IL-17 treatment promotes *in vitro* conversion of human hepatic progenitor cell line HepaRG into CSC phenotype by undergoing their **A)** dedifferentiation through reducing expression of the hepatocyte markers Albumin and HNF4α and **B)** acquisition of stem cell markers CD133 and EpCAM

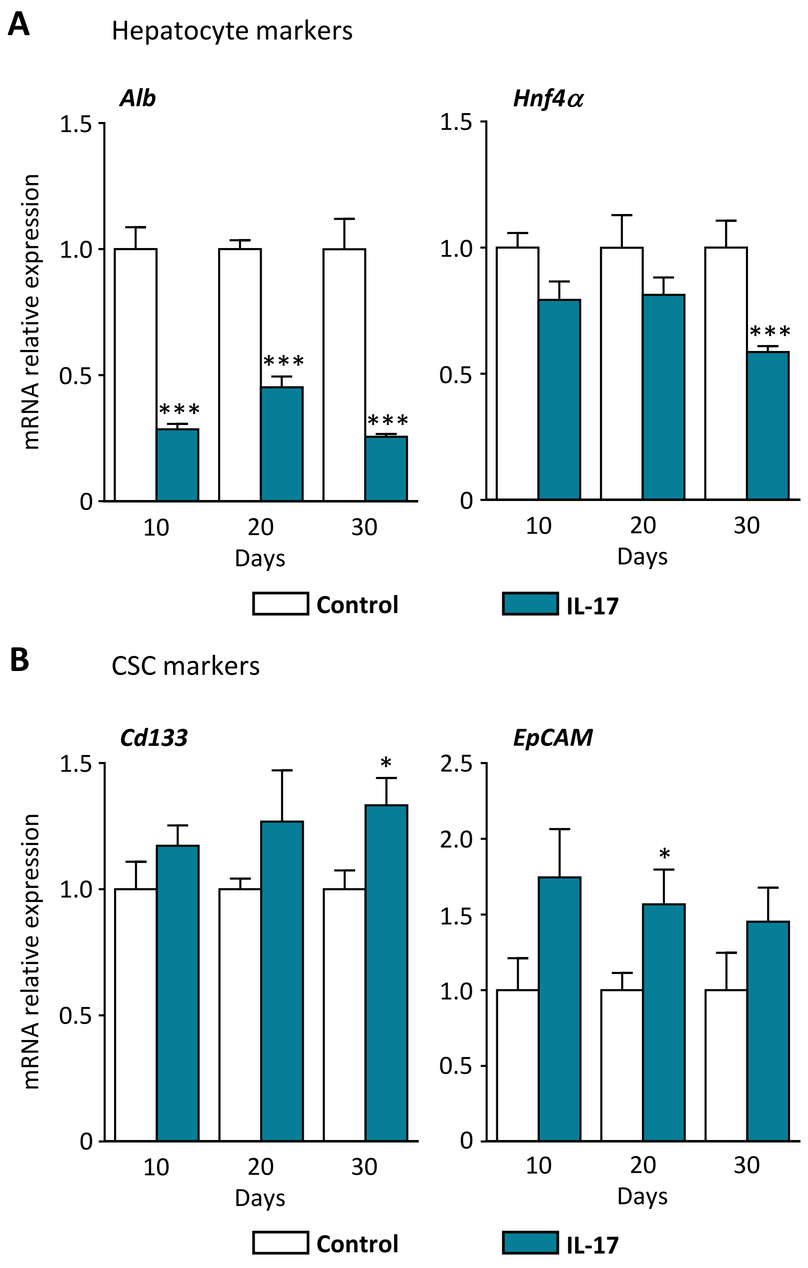

**Supplemental Figure 2:** Validation of miR-122 expression after LPC transfection with miR-122 mimics

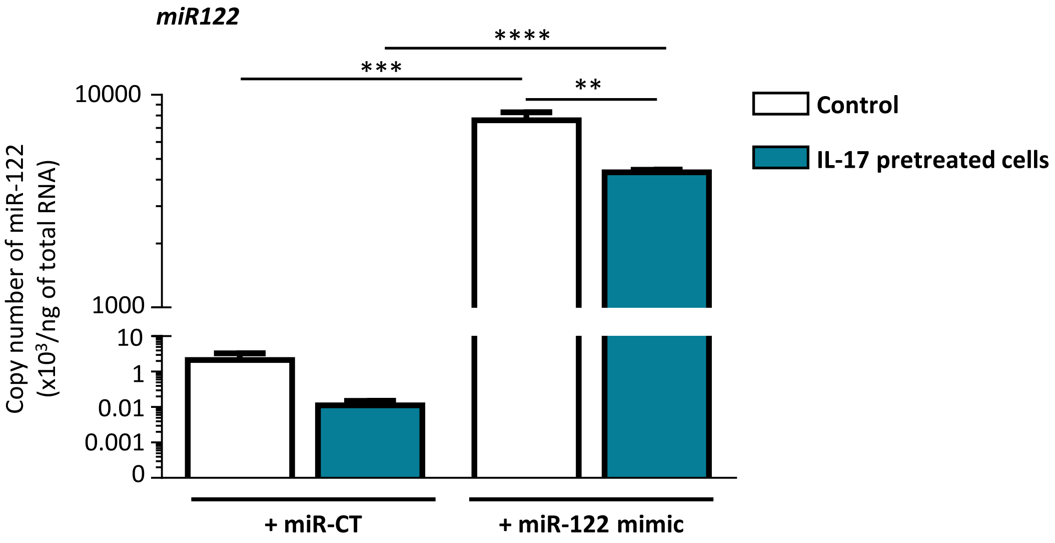

**Supplemental Figure 3 :** Stable-IL-17 transfection in LPC leads to their *in vitro* transformation into CSC phenotype thus rendering these transfected cells suitable for *in vivo* subcutaneous tumor generation. **A**) qPCR analyses showing induction of stem cell CD133 and tumor Glypican 3 expression. **B**) Flow cytometric analysis revealing induction of $CD133^{+}$ cells in LPC population and **C**) IL-17 production in transfected cell culture supernatant

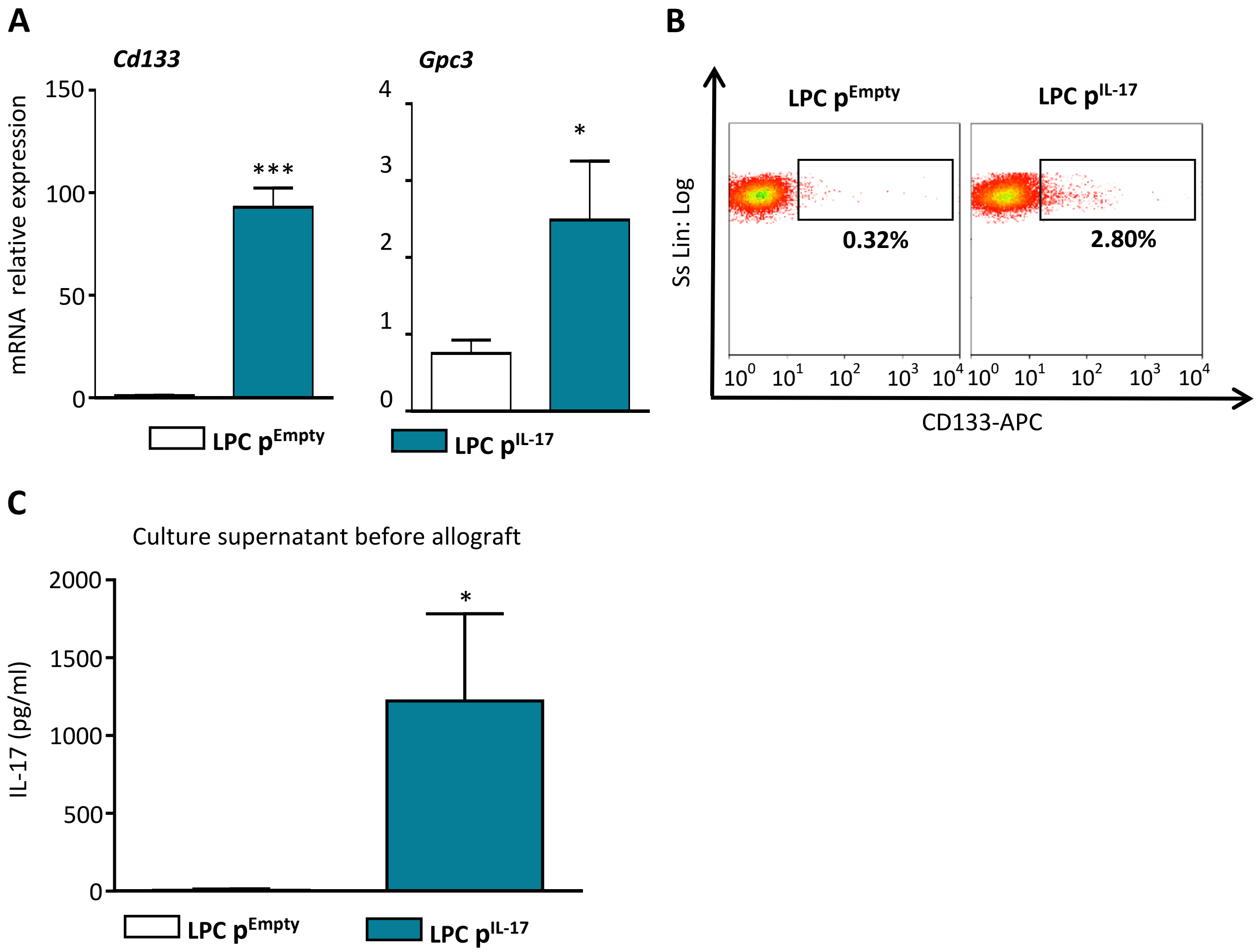

Supplement: Supplementary file 1 — Supplementary figures. [file ijbsv18p1944s1.pdf]
